# Dietary Tryptophan Plays a Role as an Anti-Inflammatory Agent in European Seabass (*Dicentrarchus labrax*) Juveniles during Chronic Inflammation

**DOI:** 10.3390/biology13050309

**Published:** 2024-04-29

**Authors:** Rita Azeredo, Diogo Peixoto, Paulo Santos, Inês Duarte, Ana Ricardo, Cláudia Aragão, Marina Machado, Benjamín Costas

**Affiliations:** 1 Centro Interdisciplinar de Investigação Marinha e Ambiental (CIIMAR), 4450-208 Matosinhos, Portugalmcasimiro@ciimar.up.pt (M.M.); 2 Instituto de Ciências Biomédicas Abel Salazar (ICBAS), Universidade do Porto, 4050-313 Porto, Portugal; 3 Centro de Ciências do Mar (CCMAR), 8005-139 Faro, Portugal; 4 Campus da Gambelas, Universidade do Algarve, 8005-139 Faro, Portugal

**Keywords:** functional ingredient, serotonergic activity, innate immunity, inflammation

## Abstract

**Simple Summary:**

Where teleost fish are concerned, the effects of a dietary tryptophan surplus are mostly immunosuppressive, making it a potential dietary anti-inflammatory strategy. The goal of the present work was to evaluate the effects of tryptophan dietary supplementation on immune and neuroendocrine responses of the European seabass, *Dicentrarchus labrax*, undergoing chronic inflammation. Juvenile European seabass were intraperitoneally injected with an inflammatory agent (inflamed group) or a saline solution (control group). Within each group, fish were fed a control and a control-based diet supplemented with tryptophan for 4 weeks. Different tissues were sampled every week for the assessment of immune-related parameters. When tryptophan was provided to fish undergoing inflammation, the gene expression of a macrophage marker increased sooner and remained high until the end of the experiment. The same fish showed a concurrent increase in peripheral monocyte counts. After one week, molecular patterns of anti-inflammatory processes seemed to be favoured by tryptophan. Altogether, results show that a short administration period seems to be critical where tryptophan supplementation is concerned since at later inflammatory stages—and longer feeding periods—fish fed this diet displayed a molecular profile similar to that of fish fed a control diet.

**Abstract:**

Where teleost fish are concerned, studies in tryptophan immunomodulation generally point to immunosuppressive properties, thus presenting a potential anti-inflammatory dietary strategy. The goal of the present work was to evaluate the effects of tryptophan dietary supplementation on immune and neuroendocrine responses of the European seabass, *Dicentrarchus labrax*, undergoing chronic inflammation. Juvenile European seabass were intraperitoneally injected with either Freund’s Incomplete Adjuvant (FIA, inflamed group) or a saline solution (control group). Within each group, fish were fed a control (CTRL) and a CTRL-based diet supplemented with tryptophan (0.3% DM basis; TRP) for 4 weeks. Different tissues were sampled every week for the assessment of immune-related parameters. When TRP was provided to FIA-injected fish, *mcsfr* gene expression increased from 1 to 2 weeks and remained high until the end of the experiment. The same fish showed a concurrent increase in peripheral monocyte counts. Moreover, *il34* expression at 1 week post-FIA injection was higher in TRP-fed than in CTRL-fed fish. After one week, molecular patterns of anti-inflammatory processes seemed to be favoured by TRP (*mcsfr*, *gr1*, *il34* and *tgfβ*). Altogether, the results show that the feeding period seems to be critical where tryptophan supplementation is concerned since at later inflammatory stages—and longer feeding periods—fish fed TRP displayed a molecular profile similar to that of the CTRL group. In contrast, shorter administration periods might accelerate immune regulatory pathways.

## 1. Introduction

Intensification of aquaculture production is inevitably accompanied by negative impacts on fish health and welfare. Current adopted rearing conditions easily increase fish susceptibility to disease, either directly—factors favouring disease outbreaks—or indirectly—inflicting stress that undermines fish immune defences [1,2]. External factors that are prolonged in time (e.g., high rearing densities, water temperature) are especially critical as they are perceived by fish as chronic stressors that eventually compromise the efficiency of fish immune responses [3]. Chronic stress is characterised by sustained intermediate cortisol levels, which trigger the reallocation of energy sources. In such conditions, several diseases may occur in less lethal forms by evolving to a chronic version, prolonged in time but inducing lower mortalities than acute episodes. Homeostasis is disrupted by energy and nutritional resources being deviated from growth, reproductive system and other physiological processes to fuel the immune system. Indeed, any stressful event will change the requirements of most nutrients [4,5]. Many essential amino acids play pivotal roles in fish immune function [6], and several studies have been conducted to investigate their potential use as feed additives to enhance fish immunity [7,8,9,10]. Tryptophan metabolism in immune cells, initiated by the indoleamine 2,3-dioxygenase (IDO), is triggered by immune stimuli such as IFNγ, LPS and TNFα, which are inducers of IDO gene expression [11,12]. This conditional metabolic fate is, therefore, exclusive of immune activation scenarios and highlights the importance of tryptophan during immune responses, as extensively studied in mammals [13,14,15]. In fish, the existence of the extra-hepatic IDO has only been officially reported in a few species [11,12], but it seems to be a conserved trait in lower vertebrates [16]. IDO activation is mostly an antimicrobial mechanism that depletes extracellular tryptophan and renders immunoregulatory metabolites (kynurenines) [17]. Despite the limited number of studies testing the effects of tryptophan dietary supplementation on fish immune responses, there is a clear trend for immunosuppression among the results obtained. Under control rearing conditions, Senegalese sole, *Solea senegalensis*, fed tryptophan-supplemented diets showed lower activity of the alternative complement pathway and lower disease resistance against infection with *Photobacterium damselae piscicida* [18]. In the European seabass, *Dicentrarchus labrax*, a tryptophan surplus inhibited the peripheral cellular immune response against *P. damselae piscicida*, as observed by decreased numbers of circulating monocytes and lymphocytes and higher susceptibility to disease [8].

Tryptophan can also regulate the immune response indirectly by modulating the hypothalamus–pituitary–interrenal axis. Tryptophan is converted into serotonin, the synthesis of which increases with increasing levels of its precursor. Interestingly, serotonin has both a stimulatory and an inhibitory effect on the stress response, which seems to be dependent on different neuroendocrine status [19,20,21]. Given the interdependency of immune and neuroendocrine systems, an extra supply of tryptophan might also indirectly affect immune mechanisms by inducing/attenuating a stress response. As previously observed, Senegalese sole reared under stressful conditions (high rearing density) and fed tryptophan-supplemented diets were better prepared to cope with a bacterial challenge (i.e., higher activity of the alternative complement pathway) and showed higher disease resistance compared to fish fed the same diet but held at control rearing conditions [18].

European seabass has long been a model species in a vast number of studies on farmed fish health and welfare, including inflammatory settings [22,23,24], and is the fish species elected for the present approach. While it might be useful to enhance immune defences a priori using a functional additive as a prophylactic strategy, fuelling a chronic inflammatory process by feeding fish immune-enhancing diets might be counterproductive. Tryptophan, however, by presenting the abovementioned properties, poses as a potential regulator of an ongoing inflammatory process and, therefore, a potential therapeutic, non-chemical strategy. Based on this hypothesis, this study’s main goal was to evaluate the effects of tryptophan dietary supplementation on immune and neuroendocrine responses of the European seabass undergoing a chronic inflammatory response.

## 2. Material and Methods

### 2.1. Experimental Dietary Treatments

Two diets were formulated and manufactured by Sparos Lda. (Olhão, Portugal). A non-supplemented diet was used as a control diet (CTRL), meeting the dietary amino acid requirements of European seabass. The supplemented diet was a CTRL-based diet supplemented with 0.3% L-tryptophan (feed weight) at the expense of wheat meal. Diets were manufactured, and amino acid content, including tryptophan, was analysed, as described by Machado and co-workers [25]. Formulation, proximate analysis and amino acid profile of the experimental diets are presented in Table 1 and Table 2, respectively.

### 2.2. Fish and Experimental Design

All experimentation performed was subjected to an ethical review process by CIIMAR’s Ethical Committee along with the CIIMAR’s Animal Welfare Body (ORBEA) in compliance with the European Directive 2010/63/EU on the protection of animals used for scientific purposes and its transposition to the Portuguese law. The experiments were conducted under the supervision of accredited researchers in laboratory animal science by the Portuguese Veterinary Authority following FELASA category C recommendations.

A total of 192 juvenile European seabass (34.55 ± 7.84 g) were randomly distributed into 12 tanks of a seawater recirculating system (n = 16; photoperiod 12 h light/12 h dark) and were acclimatised for one week being fed CTRL diet. Physicochemical parameters such as oxygen saturation (7.5 ± 0.5 mg L^−1^), salinity (27.6 ± 3.0), temperature (19.4 ± 0.9 °C) and nitrite levels (0.4 ± 0.1 mg L^−1^) were recorded daily and kept constant throughout the entire trial period. At the onset of the feeding trial, half of the fish were intraperitoneally (i.p.) injected with 100 μL of Freund’s Incomplete Adjuvant (FIA) to induce inflammation [26], while the other half was i.p. injected with 100 μL of Hanks’ Balanced Salt Solution (HBSS) to serve as a sham group. Fish were reallocated to their original tanks, and the experimental diets were randomly attributed to triplicate tanks in each group (FIA or HBSS). The feeding trial lasted for 28 days, and fish were fed manually twice a day at a daily average ration of 2% of body weight. Fish were sampled every 7 days (n = 3 per tank, n = 9 per treatment) after being euthanised by an overdose of anaesthetic (1 mL L^−1^, 2-phenoxyethanol; Merck, Germany). Blood was first withdrawn from the caudal vein with heparinised syringes and, after being used for the assessment of the haematological profile, was centrifuged for 10 min at 10,000× *g* and plasma stored at −80 °C until further processed. In addition, peritoneal cell collection was performed according to the procedure described by Afonso and co-workers [27]. The anterior gut was collected, as well as the head kidney, and both were immediately stored at −80 °C.

### 2.3. Haematological Profile

The haematological profile was assessed according to Machado et al. [28] by analysing the haematocrit, haemoglobin content (SPINREACT, Girona, Spain), and total peripheral white blood cell (WBC) and red blood cell (RBC) counts. Mean corpuscular volume (MCV), mean corpuscular haemoglobin (MCH) and mean corpuscular haemoglobin concentration (MCHC) were calculated based on the haematocrit and haemoglobin values. Blood smears were produced from fresh blood samples. Smears were firstly fixed with formol–ethanol (10% of 37% formaldehyde in absolute ethanol) and afterward stained with Wright’s stain (Haemacolor; Merck, Rahway, NJ, USA). Neutrophils were identified according to their peroxidase activity, which was detected using the method described by Afonso et al. [29]. The slides were examined under oil immersion (×1000), and at least 200 leucocytes were counted and classified as thrombocytes, lymphocytes, monocytes and neutrophils. Each cell type’s absolute concentration and relative proportion were subsequently calculated.

### 2.4. Differential Peritoneal Leucocyte Counts

Cytospin preparations of peritoneal leucocyte populations were obtained by centrifuging peritoneal cell solutions in a cellspin (THARMAC, Limburg an der Lahn, Germany). Slides were stained as previously described for blood smears. Lymphocytes, macrophages and neutrophils were identified and counted, and the percentage of each cell type was established after counting a minimum of 200 cells per slide. The concentration (×10^4^ mL^−1^) of each leucocyte type was also calculated.

### 2.5. Humoral Parameters

Lysozyme concentration was evaluated in plasma samples according to Costas et al. [30]. Total peroxidase activity was measured following the protocol described by Quade and Roth [31] and plasma bactericidal activity was determined as described by Graham and Secombes [32] with modifications [28]. Plasma cortisol was assessed by an ELISA Kit (IBL International GmbH, Hamburg, Germany), previously validated for European seabass [33] and following the manufacturer’s instructions.

### 2.6. Gut Oxidative Stress

#### Tissue Samples Homogenisation and Protein Quantification

Seabass gut samples were weighed and homogenised individually, as described by Peixoto et al. [34]. Total protein concentration in tissue homogenates was measured by using the Pierce BCA Protein Assay Kit (Rockford, IL, USA), as described by Costas et al. [35] and Machado et al. [36].

Superoxide dismutase (SOD) activity was determined following the protocols described by Almeida et al. [37] and Lima et al. [38]. Reduced/oxidised glutathione ratio (GSH/GSSG) was measured by using Microplate Assay for GSH/GSSG Kit (Oxford Biomedical Research, Rochester Hills, MI, USA), as described by Hamre et al. [39] and Tietze [40]. Catalase activity (CAT) was determined by measuring the decrease in hydrogen peroxide (H_2_O_2_) concentration as described by Claiborne [41] adapted to microplate [42].

### 2.7. Gut Immune Parameters

Gut lysozyme content, peroxidase and total bactericidal activities were evaluated following the same protocols used for plasma samples.

### 2.8. Head Kindey Gene Expression

Head kidney total RNA isolation was conducted with the NZY Total RNA Isolation kit (NZYTech, Lisbon, Portugal) following the manufacturer’s specifications. RNA was quantified using the DS-11 Spectrophotometer (DeNovix), and first-strand cDNA was synthesised with NZY First-Strand cDNA Synthesis Kit (NZYTech, Lisbon, Portugal). Quantitative PCR assays were performed with CFX384 Touch Real-Time PCR Detection System, using 4.4 μL of diluted cDNA mixed with 5 μL of NZYSpeedy qPCR Green Master Mix^®^ and 0.3 μL (10 μM) of each specific primer in a final volume of 10 μL. The cDNA amplification was carried out with specific primers for genes that were selected for their involvement in the immune response and tryptophan-related mechanisms (Table 3). Primers were designed with NCBI Primer Blast Tool and IDT OligoAnalyzer ToolTM, respecting known qPCR restrictions (amplicon size, Tm difference between primers, GC content, and self-dimer or crossdimer formation). Their efficiency was analysed in serial, 2-fold dilutions of cDNA by calculating the slope of the regression line of the cycle thresholds (Ct) vs. the relative concentration of cDNA. Melting curve analysis was also performed to verify that no primer dimers were amplified. The standard cycling conditions were 95 °C initial denaturation for 10 min, followed by 40 cycles of two steps (95 °C denaturation for 15 s followed by primer annealing temperature for 1 min), 95 °C for 1 min followed by 35 s at the annealing temperature, and finally, 95 °C for 15 s. All reactions were carried out as technical duplicates. The expression of the target genes was normalised using the geometric mean of European seabass ribosome 40 s subunit (40 s) and elongation factor 1α (*ef1α*) expression levels and calculated according to the Pfaffl method [43].

### 2.9. Statistical Analysis

Data are expressed as mean ± standard deviation (mean ± SD). Except for gene expression analysis, in which 9 individuals per treatment were used, all the remaining analyses were performed in 6 individuals (n = 6 per treatment). Data were analysed for normality and homogeneity of variance, and, when necessary, outliers were removed, and data were log-transformed before being treated statistically. Stimulus-, diet- and sampling time-induced effects were identified using a multifactorial ANOVA. When statistical significance was detected, ANOVA analyses were followed by the Tukey post hoc test to identify differences within experimental treatments. These statistical analyses were performed using the computer package Statistica 13 for Windows. The level of significance used was *p* ≤ 0.05 for all statistical tests. In an attempt to discriminate and characterise the different groups undergoing a chronic inflammatory response, a multivariate canonical discriminant analysis was performed. Thereby, numerous combinations of the original variables (discriminant functions) were evaluated. Each discriminant function explains part of the total variance of the dataset and is loaded by variables contributing the most to that variation. Wilk’s λ test assessed discriminatory effectiveness, and the distance between group centroids was measured by squared Mahalanobis distance. To attest whether these distances were statistically significant, Fisher’s F statistic was performed. Discriminant analyses were carried out using the data analysis tool XLSTAT for Microsoft Office Excel, and a significance level of 95% (*p* ≤ 0.05) was used.

## 3. Results

### 3.1. Haematological Profile

The full set of results is presented in Tables S1 and S2 of the Supplementary File. Regarding European seabass fed with the CTRL diet, total circulating WBC numbers were higher in FIA-injected fish than in the sham group at 2 weeks. The same trend was observed in their counterparts fed the TRP diet at both 2 and 4 weeks. Moreover, TRP-fed fish undergoing inflammation had higher numbers of peripheral WBC than their CTRL counterparts after 4 weeks of feeding. Amongst the TRP group, fish injected with FIA and sampled at 2 and 4 weeks showed higher WBC counts than those sampled at 3 weeks. Total RBC was lower at 2 weeks than at 1 week but gradually increased, peaking at 4 weeks, regardless of dietary treatment or stimulation. The haematocrit was lower in seabass-fed TRP compared to those fed CTRL regardless of sampling time or stimulation, as it was lower in HBSS- than in FIA-injected fish regardless of dietary treatment and sampling time, but no interactive effects were observed. Fish fed either TRP or CTRL dietary treatments showed higher haemoglobin content at 2 weeks compared to their counterparts sampled at any other sampling point, regardless of stimulation. At this particular timepoint (2 weeks), haemoglobin was higher in the TRP-fed group than in those fed CTRL. MCV was highest in seabass sampled at 2 weeks fed either diet, while MCH and MCHC were highest at 2 weeks, regardless of dietary treatment or stimulation. MCHC index was higher in TRP-fed fish than in CTRL-fed fish.

**Figure 1:**
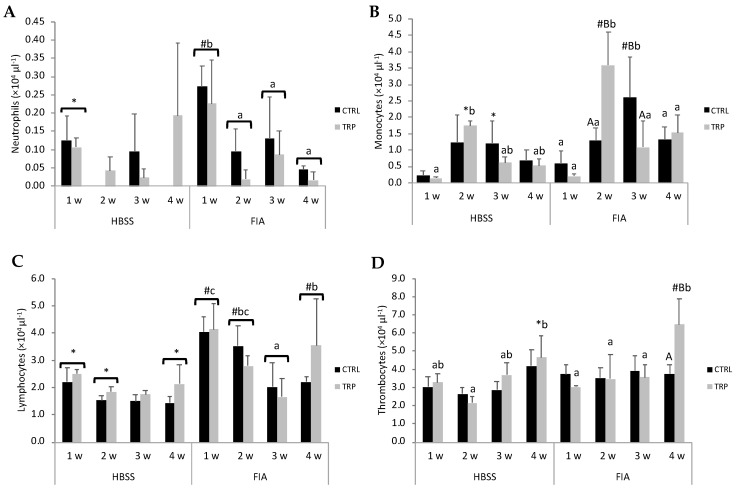
Peripheral neutrophils (**A**), monocytes (**B**), lymphocytes (**C**) and thrombocytes concentrations (**D**) in European seabass at 1, 2, 3 and 4 weeks following intraperitoneal injection with HBSS or FIA (means ± SD, n = 9). Capital letters stand for significant differences attributed to dietary treatment (A < B); lowercase letters indicate significant differences attributed to sampling time (a < b); different symbols stand for significant differences between stimuli (* < #); (multifactorial ANOVA followed by Tukey post hoc test to identify significantly different groups; *p* ≤ 0.05).

Peripheral neutrophils in FIA-injected fish were highest at 1 week, compared to the other sampling times, at which point neutrophil concentration was also higher than that measured in the HBSS group (Figure 1A). Neutrophil concentration was also highest at 1 week within each dietary treatment, CTRL and TRP. Also, a significant increase in FIA-injected fish, as opposed to the HBSS group, was only observed in the CTRL group and not in TRP. An increase followed by a subsequent drop in peripheral monocyte numbers was observed in FIA-injected fish in both dietary treatments (Figure 1B); yet, while the highest concentration in the CTRL group was observed at 3 weeks, in fish fed the TRP diet, it was detected at 2 weeks. At both peaking points, FIA-injected fish showed significantly higher monocyte numbers than their HBSS counterparts. No effects of dietary treatments were observed on lymphocyte numbers (Figure 1C). FIA-injected fish sampled at 1 and 2 weeks presented higher lymphocyte concentrations than those injected with HBSS. Additionally, peripheral lymphocyte counts of fish undergoing inflammation were lowest at 3 weeks, and despite it increasing at 4 weeks, levels were still lower than those of fish sampled at 1 week. Thrombocyte concentration at 4 weeks in TRP, FIA-injected group was the highest amongst all comparable groups (Figure 1D).

### 3.2. Peritoneal Leucocytes

The full set of results is presented in Table S3 of the Supplementary File.

**Figure 2:**
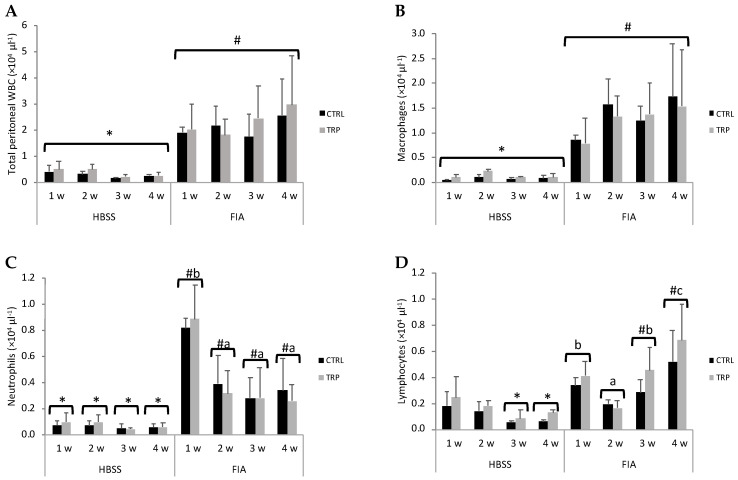
Total WBC (**A**), macrophages (**B**), neutrophils (**C**) and lymphocyte concentrations (**D**) in the peritoneal cavity of European seabass at 1, 2, 3 and 4 weeks following intraperitoneal injection with HBSS or FIA (means ± SD, n = 9). Lowercase letters indicate significant differences attributed to sampling time (a < b); different symbols stand for significant differences between stimuli (* < #); (multifactorial ANOVA followed by Tukey post hoc test to identify significantly different groups; *p* ≤ 0.05).

Total peritoneal WBC and macrophages were not significantly affected by dietary treatment or sampling time, but counts were higher in FIA- than in HBSS-injected fish (Figure 2A and Figure 2B, respectively). Neutrophils were more abundant at all timepoints in fish undergoing inflammation than in their sham counterparts, while the same pattern was observed regarding lymphocyte concentration at 3 and 4 weeks (Figure 2C). In FIA-injected fish, neutrophils were higher at 1 week compared to the remaining sampling points. Regarding lymphocytes, inflamed fish showed an initial decrease from week 1 to week 2, but numbers significantly and gradually increased, peaking at week 4 (Figure 2D). No effects of dietary treatments were observed on any peritoneal cell type.

### 3.3. Plasma Cortisol and Immune Parameters

**Figure 3:**
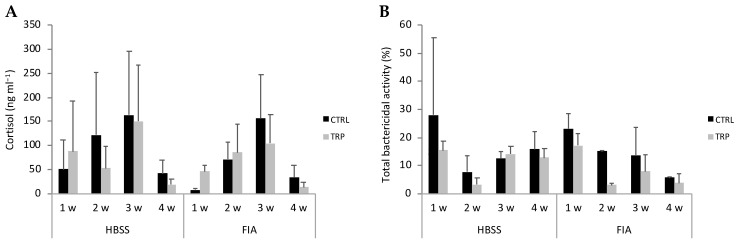
Cortisol (**A**) and total bactericidal activity (**B**) in plasma of European seabass before (0 h) or at 1, 2, 3 and 4 weeks following intraperitoneal injection with HBSS or FIA (means ± SD, n = 9). multifactorial ANOVA followed by Tukey post hoc test to identify significantly different groups (*p* ≤ 0.05).

The full set of results is presented in Table S4 of the Supplementary File. Plasma cortisol levels and peroxidase activity were modulated by sampling time, regardless of dietary treatment or stimulation (Figure 3A). Cortisol peaked at 3 weeks and then dropped to levels similar to those observed at 1 week. Differently, peroxidase activity in fish sampled at 4 weeks was lower than in those sampled at 1 week. Lysozyme concentration in FIA-injected fish increased over time and was higher than HBSS-injected fish at 4 weeks. Total bactericidal activity decreased from 1 to 2 weeks and remained low until the last sampling point, regardless of dietary treatment and stimulation (Figure 3B). Additionally, the TRP group’s bactericidal activity was lower than that of the CTRL group, irrespective of sampling time or stimulation.

### 3.4. Gut Oxidative Stress and Immune Parameters

**Figure 4:**
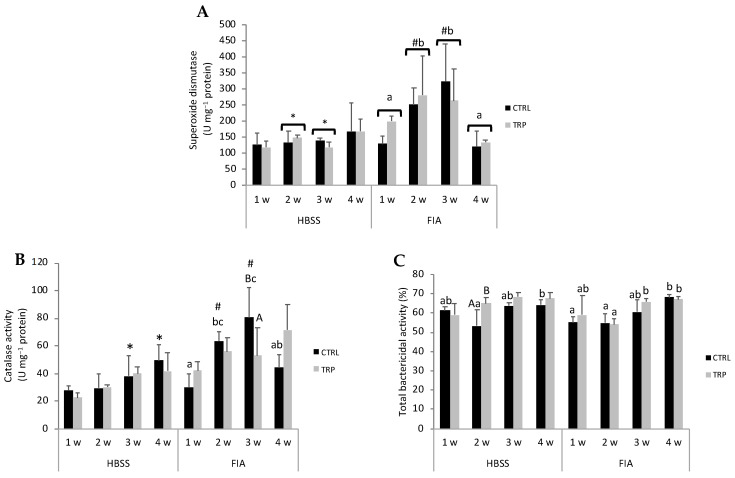
Superoxide dismutase (**A**), catalase (**B**) and total bactericidal activity (**C**) in the gut of European seabass before (0 h) or at 1, 2, 3 and 4 weeks following intraperitoneal injection with HBSS or FIA (means ± SD, n = 9). Capital letters stand for significant differences attributed to dietary treatment (A < B); lowercase letters indicate significant differences attributed to sampling time (a < b); different symbols stand for significant differences between stimuli (* < #); (multifactorial ANOVA followed by Tukey post hoc test to identify significantly different groups; *p* ≤ 0.05).

The full set of results is presented in Table S5 of the Supplementary File. The activity of gut superoxide dismutase increased from 1 to 2 weeks and then dropped at 4 weeks to levels similar to those of the first sampling point (Figure 4A). Its activity was higher in FIA-injected fish than in HBSS-injected ones at 2 and 3 weeks. Catalase activity in the gut of FIA-injected fish fed the CTRL diet gradually increased and peaked at 3 weeks, followed by a decrease at 4 weeks to levels similar to those observed at 1 week (Figure 4B). Among fish fed the CTRL diet, catalase activity was higher in inflamed than in sham fish at 2 and 3 weeks, at which timepoint it was also observed to be higher in CTRL, FIA-injected fish than in their TRP counterparts. The GSH/GSSG ratio was lower in inflamed fish than in the sham group, regardless of dietary treatment, but only at 2 weeks.

Peroxidase activity was lower in fish injected with FIA than in those injected with HBSS and momentarily decreased at 2 weeks post-injection, but no effects of dietary treatment were noted. The gut of HBSS-injected fish fed TRP had higher total bactericidal activity than those fed a CTRL diet 2 weeks post-injection (Figure 4C). Additionally, CTRL-fed fish undergoing inflammation showed a gradual increase in gut bactericidal activity that peaked at 4 weeks, while those fed TRP did not show a similar trend.

### 3.5. Gene Expression

For a clearer view of the gene expression profile, results were classified as inflammation-induced changes (i.e., changes attributed to the inflammatory response elicited by FIA i.p. injection, irrespective of dietary treatment) and dietary treatment-induced changes associated or not with the inflammatory response. The complete set of results is presented in Table S6 of the Supplementary File.

#### 3.5.1. Inflammation-Induced Changes

**Figure 5:**
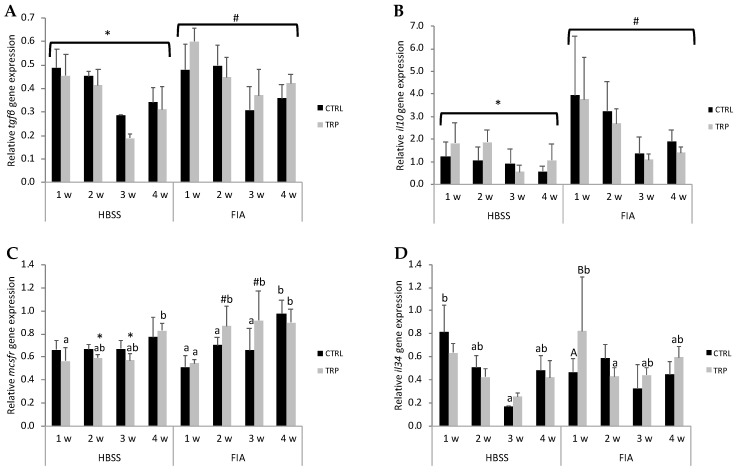
Gene expression of transforming growth factor β (**A**, *tgfβ*), interleukin 10 (**B**, *il10*), macrophage colony-stimulating factor receptor (**C**, *mcsfr*) and interleukin 34 (**D**, *il34)* in the head kidney of European seabass before (0 h) or at 1, 2, 3 and 4 weeks following intraperitoneal injection with HBSS or FIA (means ± SD, n = 9). Capital letters stand for significant differences attributed to dietary treatment (A < B); lowercase letters indicate significant differences attributed to sampling time (a < b); different symbols stand for significant differences between stimuli (* < #); (multifactorial ANOVA followed by Tukey post hoc test to identify significantly different groups; *p* ≤ 0.05).

Fish i.p. injected with FIA showed lower expression of *gr1* compared to HBSS-injected fish (at 1 week, irrespectively of dietary treatment), as well as a downregulation of *il1β* from 1 to 3 weeks and of *cxcr4* from 1 to 4 weeks in those fed CTRL. In contrast, *tgfβ* and *il10* were higher in FIA-injected fish than in HBSS counterparts, regardless of dietary treatment and sampling point (Figure 5A and Figure 5B, respectively). The expression of *mcsfr* was enhanced in inflamed fish fed CTRL from 3 to 4 weeks post-injection (Figure 5C).

#### 3.5.2. Dietary Treatment-Induced Changes

In the group injected with HBSS and fed CTRL, the expression of *il34* was downregulated from 1 to 3 weeks (Figure 5D), while the expression of *il1β* increased from 3 to 4 weeks. Regarding those fed TRP, *mcsfr* increased over time with higher expression at 4 weeks than at 1 week post-injection (Figure 5C), and *cxcr4* was significantly lower at 3 weeks compared to CTRL counterparts.

In fish undergoing an inflammatory response (FIA-injected fish), TRP-fed fish showed upregulation of *mcsfr* from 1 to 2 weeks, and expression remained high until the end of the experiment (Figure 5C). Additionally, at 2- and 3-week sampling points, these fish showed higher *mcsfr* expression levels than their HBSS counterparts. These differences were absent in CTRL-fed fish, in which *mcsfr* mRNA levels increased only from 3 to 4 weeks. Moreover, *il34* mRNA levels at 1 week post-FIA injection were higher in TRP-fed than in CTRL-fed fish, but the expression was downregulated to levels similar to CTRL at 2 weeks (Figure 5D).

Both groups fed CTRL and TRP dietary treatments showed a time-dependent downregulation of *cxcr4* during the inflammatory response. No effects of inflammation nor dietary treatments were observed in both *tcr* and *ido2* expression profiles. A significant time-dependent decrease was noted in *mc2r* gene expression, regardless of dietary treatment or stimulation.

**Figure 6:**
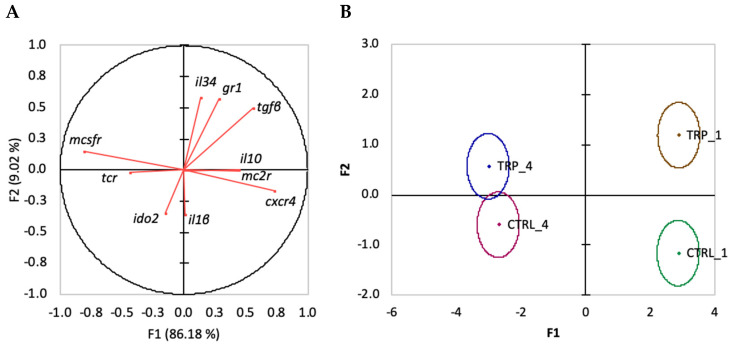
Canonical discriminant analysis of gene expression in the head kidney of European seabass sampled at one or four weeks following intra-peritoneal injection with FIA: (**A**) correlation variables/factors (factor loads) for two main discriminant functions (F1 and F2); (**B**) canonical discriminant scores of each group. Group centroids are marked by a small diamond, whereas circles indicate data distribution per group.

When evaluating linear functions of variables (immune-related transcripts in the head kidney) and their contributions to differences between dietary groups undergoing an inflammatory response for one and for four weeks (CTRL_1, CTRL_4, TRP_1 and TRP_4), the overall discriminant analysis performance was very reasonable (Wilks λ = 0.033, *p* < 0.0001). It resulted in three discriminant functions, with the first two accounting for 95.2% of the total variability (Figure 6A). The first discriminant function (F1, 86.2%) was negatively loaded by *mcsfr* (i.e., lower gene expression) and positively loaded by *cxcr4* and *tgfβ* (i.e., higher gene expression; Figure 6A, correlations of −0.81, 0.73 and 0.56, respectively), whereas the second function (F2, 9%) was positively loaded by *il34* and *gr1* (Figure 6A, correlations of 0.58 and 0.57, respectively). The analysis of Mahalanobis distances between groups’ multivariate means demonstrated that CTRL_1 is significantly different from CTRL_4, and TRP_1 is significantly different from TRP_4 (*p* < 0.0001, Figure 6B).

## 4. Discussion

A chronic inflammation model was carried out for four weeks by means of an intra-peritoneal injection with FIA. Since it is a mixture of oil and water, its absorbance/degradation by the host takes a while, but it poses no further pathological damage and risks [26]. Since the adjuvant is perceived as a foreign body upon injection, it can trigger the host’s innate immune mechanisms, thereby eliciting an inflammatory response. Like any other immune process, and whatever the initial trigger is, inflammatory mechanisms are recognised in central brain structures (i.e., the hypothalamus), and neuroendocrine regulatory mechanisms are put in motion [44,45]. A rise in cortisol levels was, therefore, somewhat expected in FIA-injected fish. A comparable increase in this stress hormone would also be accepted in HBSS-injected fish fed TRP, as previously observed in non-stimulated rainbow trout, *Oncorhynchus mykiss* [19] and even European seabass [8]. Unexpectedly, all fish from the present study experienced a transversal, gradual increase in cortisol levels that peaked at the end of the third week. Nonetheless, cortisol is a very accurate stress parameter at an early phase of the neuroendocrine response (acute phase) [1]. In a similar future approach, it would be even more interesting to look at plasma glucose levels, which are generally a better indicator of the secondary phase and, thus, perhaps more appropriate in chronic stressful situations. It seems, however, that fish were finally able to cope with their context after four weeks when cortisol levels resembled those of resting fish. In parallel, the expression of ACTH receptor, *mc2r*, which activation signals for cortisol secretion as well as that of cortisol receptor itself (*gr1*), gradually and transversally decreased until the third week, possibly as a counteracting strategy. This increase might have masked a potential modulatory effect of both TRP and FIA in the hypothalamus–pituitary–interrenal axis, usually reflected in the rise/fall of cortisol levels. Irrespective of the factor triggering its release, plasma cortisol fluctuations in time seem to be related to temporal changes in several genes’ expression profiles, regardless of dietary treatment or injection nature. Similar to what is known in mammals, the majority of cortisol effects in fish are known to be mediated by its receptors’ transactivation and transrepression activities in what is known as the genomic pathway [46,47]. Hence, it is not surprising that the expression of these immune-related genes fluctuates in time in parallel with cortisol temporal changes.

Despite this apparent cortisol suppressive effect on gene expression, the injection with FIA was able to induce a clear, robust inflammatory response reflected in both molecular and humoral levels. FIA-injected fish were characterised for having higher mRNA expression levels of anti-inflammatory cytokines (IL10 and TGFβ), which typically proliferate in later stages of inflammatory reactions, coordinating regulatory effects [48]. In addition, though transversally downregulated in injected fish compared to undisturbed fish, gene expression of the pro-inflammatory IL1β was further repressed in inflamed fish three weeks post FIA challenge, while no such inhibition was observed in those injected with HBSS. Similar results were reported in a previous study using a European seabass chronic inflammatory model, in which gene expression of pro-inflammatory cytokines, such as IL1β, were found downregulated at later stages of the response (three weeks post FIA i.p. injection) [49]. Besides these evident changes at the molecular level, humoral and cellular defences were also modulated. Every inflammatory response is systemically supported by a wide array of humoral and cellular defences. As expected, plasma lysozyme content was found to be higher in inflamed fish, in parallel to a reinforced cellular capacity that was noted peripherally and locally (peritoneal cavity). Also, oxidative stress was intensified at the gut level, as indicators such as CAT and SOD activities were observed to be enhanced two weeks after the i.p. injection, and further supported by the lower GSH/GSSG ratio. This enhanced enzymatic activity suggests increased oxidative stress in the gut, but the inflammatory focus was not on the gastrointestinal tract. Nevertheless, a prolonged state of inflammation (even originally elsewhere) might have triggered changes at the systemic level that, in turn, led to alterations in gut homeostasis [50]. These alterations are highly dependent on each specific context since contrasting gut responses were observed in European seabass-fed tryptophan-supplemented diets for fifteen days and subjected to intra-peritoneal infection with *Photobacterium damselae piscicida* [25].

The present study was designed and performed to highlight the possible modulatory effects of tryptophan dietary supplementation on the development/resolution of a chronic inflammatory response. Tryptophan surplus caused a few changes by itself (regardless of whether fish were injected with either FIA or HBSS). Interestingly, opposite outcomes were pulled in systemic and gut general bactericidal capacity, with TRP strengthening defences in the intestine of unstimulated fish but impairing plasma-borne ones in both stimulated and non-stimulated fish. An impairment of plasma total bactericidal activity has been previously related to tryptophan dietary supplementation in European seabass-fed tryptophan-supplemented diets for fifteen days [25]. On the other hand, the stimulation of gastrointestinal immune defences by TRP could be related to one or several variables not inspected in the present experimental design. Indeed, in the context of the present study, the gastrointestinal tract was not targeted as the inflamed tissue, but it was nonetheless the first matrix to have contact with the testing factor—the amino acid tryptophan. Mediators of the gut-associated lymphoid tissue and gut microbiota itself are, therefore, prone to changes upon higher tryptophan availability. Hence, alterations in immune defences at the gut level could have been the result of upstream changes such as microbiota dysbiosis and related immune mediators’ activation and vice-versa [51,52]. In addition, and in the absence of an inflammatory process, tryptophan dietary surplus prevented the increase in *il1β* expression levels seen in fish fed CTRL four weeks after HBSS injection. The mRNA levels of this pro-inflammatory gene were particularly low, which was probably a result of high cortisol levels, as previously discussed. Once cortisol levels decreased (from three to four weeks post-injection), *il1β* significantly increased in CTRL-fed fish but remained similarly low in TRP-fed fish, adding to the immune regulatory properties of tryptophan.

When inflammation and tryptophan supplementation were concurrent, monocyte counts were much higher as soon as two weeks following inflammation. Moreover, the number of circulating leucocytes was higher with TRP at four weeks than with CTRL. The increase is particularly explained by thrombocytes that were far more numerous in the TRP-fed group than in CTRL-fed counterparts. There is no evidence that higher dietary intake of tryptophan in mammals results in a higher number of circulating platelets; in turn, platelet-released serotonin is involved in platelet aggregation and vasoconstriction [53]. Additionally, and in contrast to mammals, fish do not seem to carry serotonin reserves in thrombocytes; rather, it is transported in the extracellular plasma [54]. Nevertheless, this result relating dietary tryptophan surplus with peripheral thrombocyte numbers seems to indicate the existence of further links between the amino acid and fish thrombocytes that do not necessarily involve serotonin.

Considered a good marker of chronic inflammation [49], *mcsfr* was not particularly enhanced in this inflammatory setting, perhaps given the stressful state of the whole stock as previously suggested, but it increased over time in FIA-injected fish. Interestingly, feeding seabass TRP led to a faster increase in *mcsfr* expression levels (relative to CTRL). Interleukin 34 shares its receptor (MSCFR1) with the colony-stimulating factor (CSF), and both seem to have similar ability to induce monocyte differentiation, but IL34 was reported to more strongly induce IL10 secretion by monocytes/macrophages as compared to CSF [55]. The expression of *il34* in TRP-fed fish was twice that of CTRL fish at one week post-injection. These observations are almost coincident with the abovementioned monocytosis observed in the same fish. Together, these results suggest an expressive favouring of cellular regulatory phenotypes when fish were provided a tryptophan surplus.

When focusing on FIA-injected fish belonging to the tail groups of the present experimental design (shortest and longest feeding periods), the first discriminant function clearly differentiates groups fed dietary treatments for one week from those fed for 4 weeks. This distance between them highlights the clearly different dynamics of fish at an early phase of the response and of those at later stages. The variables that most contributed to this separation were *mcsfr* and *cxcr4*, though in opposite directions. More interestingly, dietary groups fed for 1 week were further apart from each other (more different) than those fed for 4 weeks, along the axis of the second discriminant function. Positively loaded by *gr1* and *il34*, this function thereby stresses out the importance of the feeding period in what tryptophan supplementation is concerned and points to tryptophan modulatory effects on the ability of fish to deal with cortisol high levels during inflammation (higher *gr1* expression levels), as well as on regulatory mechanisms that might accelerate inflammation resolution (higher *il34* expression levels).

## 5. Conclusions

The present study gives a new perspective on the potential use of tryptophan as a dietary additive to regulate chronic inflammatory responses in aquaculture fish species. Future analytical work for a more comprehensive approach should include the evaluation of indicators of the secondary stress response, such as plasma glucose levels and lactate. In general, results show that dietary tryptophan supplementation for four weeks did not significantly alleviate inflammatory conditions; however, peripheral cell counts and molecular analysis of head kidney immune-related gene expression provided indications that a shorter administration period might accelerate immune regulatory pathways associated with anti-inflammatory immune cells. The fact that differences are attenuated in the following weeks depicts the importance of optimising feeding periods in the application of such immune-enhancing strategies.

## Figures and Tables

**Table 1:** Ingredient and chemical composition of the experimental diets.

Ingredients (%)	CTRL	TRP
Fish protein hydrolysate ^1^	5.00	5.00
Fish gelatin ^2^	2.00	2.00
Soy protein concentrate ^3^	25.00	25.00
Pea protein concentrate ^4^	6.00	6.00
Wheat gluten ^5^	10.00	10.00
Corn gluten meal ^6^	15.00	15.00
Wheat meal ^7^	15.80	15.50
Vit and Min Premix ^8^	1.00	1.00
Antioxidant ^9^	0.20	0.20
Sodium propionate ^10^	0.10	0.10
Monocalcium phosphate ^11^	3.00	3.00
L-Lysine 99% ^12^	0.60	0.60
L-Tryptophan ^13^	0.00	0.30
DL-Methionine ^14^	0.20	0.20
Soy lecithin ^15^	1.00	1.00
Fish oil ^16^	15.10	15.10
Total	100.00	100.00
Proximate analyses (% dry weight)		
Crude protein (%)	45.70	46.00
Crude fat (%)	18.00	18.00
Fiber (%)	1.70	1.70
Starch (%)	13.40	13.20
Ash (%)	6.80	6.80
Energy (MJ kg^−1^)	21.90	21.90

^1^ CPSP 90: 82.6% crude protein (CP), 9.6% crude fat (CF), Sopropêche, France; ^2^ Fish gelatin: 88% CP, 0.1% CF, LAPI Gelatine SPA, Italy; ^3^ Soycomil P: 63% CP, 0.8% CF, ADM, The Netherlands; ^4^ NUTRALYS F85F: 78% CP, 1% CF, ROQUETTE Frères, France; ^5^ VITAL: 83.7% CP, 1.6% CF, ROQUETTE Frères, France; ^6^ Corn gluten meal: 61% CP, 6% CF, COPAM, Portugal; ^7^ Wheat meal: 10.2% CP; 1.2% CF, Casa Lanchinha, Portugal; ^8^ PREMIX Lda, Portugal: Vitamins (IU or mg kg^−1^ diet): DL-alpha tocopherol acetate, 100 mg; sodium menadione bisulphate, 25 mg; retinyl acetate, 20000 IU; DL-cholecalciferol, 2000 IU; thiamin, 30 mg; riboflavin, 30 mg; pyridoxine, 20 mg; cyanocobalamin, 0.1 mg; nicotinic acid, 200 mg; folic acid, 15 mg; ascorbic acid, 500 mg; inositol, 500 mg; biotin, 3 mg; calcium panthotenate, 100 mg; choline chloride, 1000 mg, betaine, 500 mg. Minerals (g or mg/kg diet): copper sulphate, 9 mg; ferric sulphate, 6 mg; potassium iodide, 0.5 mg; manganese oxide, 9.6 mg; sodium selenite, 0.01 mg; zinc sulphate, 7.5 mg; sodium chloride, 400 mg; excipient wheat middlings; ^9^ Paramega PX, Kemin Europe NV, Belgium; ^10^ PREMIX Lda., Portugal; ^11^ MCP: 22% phosphorus, 16% calcium, Fosfitalia, Italy; ^12^ L-Lysine HCl 99%, Ajinomoto Eurolysine SAS, France. ^13^ L-Tryptophan 98%, Ajinomoto Eurolysine SAS, France; ^14^ DL-Methionine for Aquaculture: 99% Methionine, Evonik Nutrition & Care GmbH, Germany; ^15^ Lecico P700IPM, LECICO GmbH, Germany; ^16^ SAVINOR UTS, Portugal.

**Table 2:** Amino acid composition of the experimental diets.

Amino Acids (% Dry Weight)	CTRL	TRP
Arginine	4.0	4.1
Histidine	1.2	1.2
Lysine	3.0	3.0
Threonine	1.8	1.9
Isoleucine	2.1	2.1
Leucine	4.2	4.3
Valine	2.3	2.3
Tryptophan	0.2	0.4
Methionine	1.1	1.2
Phenylalanine	3.0	3.2
Cysteine	0.4	0.4
Tyrosine	2.6	2.8
Aspartic acid	3.4	3.2
Glutamic acid	9.7	9.1
Alanine	2.4	2.5
Glycine	2.7	2.7
Proline	3.7	3.6
Serine	2.6	2.6

**Table 3:** Forward and reverse primers for real-time PCR.

Acronym	Gene	GenBank	Eff ^2^	AT ^3^	Product Length ^4^	Forward Primer Sequence	Reverse Primer Sequence
*40s*	40s Ribosomal protein	HE978789.1	105.7	60	79	TGATTGTGACAGACCCTCGTG	CACAGAGCAATGGTGGGGAT
*ef1α*	Elongation factor 1 β	AJ866727.1	97.2	57	144	AACTTCAACGCCCAGGTCAT	CTTCTTGCCAGAACGACGGT
*gr1*	Glucocorticoid receptor 1	AY619996.1	104.1	60	100	AAATCTGCCTGGTGTGTTCC	TGCCCTTTCACTGCTCTCTT
*mc2r*	Melanocortin 2 receptor	DLAgn_00065140 ^1^	101	60	118	GAGGGCAAGGGGAGCATTTA	GACGGGCAGATGGCAGTTAT
*tcr* *α*	T-cell receptor α chain	DLAgn_00260540 ^1^	105.3	60	72	ACACTGGCTGAGAAACATCCT	GGCTGGGTCACTCTGTCTTC
*ido2*	Indoleamine 2,3-dioxygenase	DLAgn_00014730 ^1^	108.1	60	74	TGAAGGTGTGAGCAATGAGC	CAAAGCACTGAATGGCTGAA
*il1* *β*	Interleukin 1 β	AJ269472.1	93.2	57	105	AGCGACATGGTGCGATTTCT	CTCCTCTGCTGTGCTGATGT
*mcsfr*	Macrophage colony-stimulating factor receptor	DLAgn_00109630 ^1^	83.0	62	200	ATGTCCCAACCAGACTTTGC	GGCTCATCACACACTTCACC
*cxcr4*	Chemokine CXC receptor 4	FN687464.1	91.7	57	171	ACCAGACCTTGTGTTTGCCA	ATGAAGCCCACCAGGATGTG
*il34*	Interleukin 34	DLAgn_00164750 ^1^	105.3	60	129	GGAAATACGCTTCAGGGATG	GGCACTCTGTCGGGTTCTT
*tgf* *β*	Transforming growth factor β	AM421619.1	97.9	55	143	ACCTACATCTGGAACGCTGA	ACCTACATCTGGAACGCTGA
*il10*	Interleukin 10	AM268529.1	112.75	55	164	ACCCCGTTCGCTTGCCA	CATCTGGTGACATCACTC

^1^ Sequences obtained from databases dicLab v1.0c seabass genome. ^2^ Efficiency of PCR reactions were calculated from serial dilutions of tissue RT reactions in the validation procedure. ^3^ Annealing temperature (°C). ^4^ Amplicon (nt).

## Data Availability

All data are provided in the main text or Supplementary Files.

## Supplementary Materials

The following supporting information can be downloaded at: https://www.mdpi.com/article/10.3390/biology13050309/s1, Table S1: Haematological parameters of European seabass fed dietary treatments and sampled at 1, 2, 3 and 4 weeks post-injection; Table S2: Peripheral leucocyte counts of European seabass fed dietary treatments and sampled at 1, 2, 3 and 4 weeks post-injection; Table S3: Peritoneal leucocyte counts of European seabass fed dietary treatments and sampled at 1, 2, 3 and 4 weeks post-injection; Table S4: Humoral parameters in European seabass fed dietary treatments and sampled at 1, 2, 3 and 4 weeks post-injection; Table S5: Immune and oxidative stress parameters in the gut of European seabass fed dietary treatments and sampled at 1, 2, 3 and 4 weeks post-injection; Table S6: Gene expression in the head kidney of European seabass fed dietary treatments and sampled at 1, 2, 3 and 4 weeks post-injection.
