# Supplementary material for: Dietary Tryptophan Plays a Role as an Anti-Inflammatory Agent in European Seabass (Dicentrarchus labrax) Juveniles during Chronic Inflammation
Source: Biology (Basel). 2024 Apr 29;13(5):309. doi: 10.3390/biology13050309 (PMC11117642; doi:10.3390/biology13050309)
Supplement: Supplementary file 1 [file biology-13-00309-s001.zip › biology-2959485-supplementary.pdf]

Dietary tryptophan plays a role as an anti-inflammatory agent in European seabass (Dicentrarchus labrax) juveniles during chronic inflammation

Azeredo, R.[1], Peixoto D.[1,2], Santos, P.[1,2], Duarte, I[1,2]. D., Ricardo[1,2], A., Aragão, C.[3,4], Machado, M.[1] and Costas, B.[1,2]

[1] Centro Interdisciplinar de Investigação Marinha e Ambiental (CIIMAR), 4450-208 Matosinhos, Portugal
[2] Instituto de Ciências Biomédicas Abel Salazar (ICBAS), Universidade do Porto, 4200-135 Porto, Portugal
[3] Centro de Ciências do Mar (CCMAR), 8005-139 Faro, Portugal
[4] Universidade do Algarve, 8005-139 Faro, Portugal

***** Correspondence: mleme@ciimar.up.pt

Supplementary File

Table S1. Haematological parameters of European seabass fed dietary treatments and sampled at 1, 2, 3 and 4 weeks post injection.

Haematological parameters	Dietary treatment	1 week		2 weeks		3 weeks		4 weeks	
		HBSS	FIA	HBSS	FIA	HBSS	FIA	HBSS	FIA
WBC ($\times 10^4$ µl^{-1})	CTRL	5.6 ± 1.1	8.7 ± 0.5	5.5 ± 0.5 *	9.5 ± 1.6 #	5.8 ± 1.0	8.9 ± 0.8	6.2 ± 1.2	8.4 ± 1.3 A
	TRP	6.2 ± 0.4	7.6 ± 1.2 ab	5.9 ± 1.0 *	10.4 ± 0.3 #bc	6.1 ± 0.3	6.0 ± 1.0 a	7.1 ± 1.9 *	11.6 ± 3.4 #Bc
RBC ($\times 10^6$ µl^{-1})	CTRL	2.6 ± 0.7	2.7 ± 0.4	2.4 ± 0.3	2.2 ± 0.1	2.7 ± 0.4	2.7 ± 0.5	2.9 ± 0.3	3.0 ± 0.4
	TRP	2.5 ± 0.2	2.6 ± 0.2	2.2 ± 0.2	2.2 ± 0.3	2.4 ± 0.3	2.7 ± 0.2	2.7 ± 0.3	3.0 ± 0.1
Haematocrit (%)	CTRL	30.8 ± 1.9	31.4 ± 1.9	31.2 ± 3.0	32.6 ± 3.3	27.7 ± 2.4	31.8 ± 1.7	27.6 ± 2.7	33.0 ± 2.3
	TRP	26.3 ± 6.0	28.2 ± 3.3	27.8 ± 5.3	31.2 ± 1.6	27.4 ± 1.1	28.2 ± 3.2	29.3 ± 4.0	33.0 ± 2.2
Haemoglobin (g dl^{-1})	CTRL	1.2 ± 0.1	1.2 ± 0.3	2.0 ± 0.2	1.9 ± 0.3	1.3 ± 0.1	1.3 ± 0.2	1.3 ± 0.2	1.4 ± 0.3
	TRP	1.2 ± 0.2	1.2 ± 0.2	2.2 ± 0.3	2.3 ± 0.3	1.3 ± 0.3	1.4 ± 0.1	1.3 ± 0.2	1.3 ± 0.2
MCV (µm^3)	CTRL	137.4 ± 39.2	118.4 ± 21.3	122.7 ± 6.6	133.4 ± 19.7	108.0 ± 14.2	125.2 ± 15.9	102.9 ± 2.9	103.6 ± 3.5
	TRP	109.5 ± 15.9	103.3 ± 10.6	122.2 ± 8.4	140.8 ± 20.8	114.9 ± 7.1	99.7 ± 14.1	107.8 ± 5.4	109.4 ± 17.3
MCH (pg cell^{-1})	CTRL	4.2 ± 0.5	4.5 ± 1.0	8.5 ± 0.6	9.2 ± 1.1	4.9 ± 1.2	4.4 ± 0.5	4.9 ± 0.2	4.6 ± 0.5
	TRP	4.5 ± 1.0	4.7 ± 1.0	8.4 ± 1.4	9.7 ± 1.3	5.7 ± 0.8	5.2 ± 0.8	4.9 ± 0.2	4.7 ± 0.6
MCHC (g 100 mL^{-1})	CTRL	3.9 ± 0.4	3.4 ± 0.3	6.5 ± 0.2	6.3 ± 0.9	4.5 ± 0.5	4.1 ± 0.7	4.6 ± 0.1	4.2 ± 0.2
	TRP	4.9 ± 1.9	4.4 ± 1.0	7.6 ± 2.1	7.3 ± 0.6	5.0 ± 0.5	4.8 ± 0.8	4.5 ± 0.7	4.4 ± 0.9

Factorial ANOVA	P-value							Time				Stimulus		Diet	
Haematological parameters	Time	Stimulus	Diet	Time × Stimulus	Time × Diet	Diet × Stimulus	Time × Diet × Stimulus	1w	2w	3w	4w	HBSS	FIA	CTRL	TRP
WBC	0.002	< 0.001	ns	0.02	0.003	ns	0.02								
RBC	< 0.001	ns	ns	ns	ns	ns	ns	b	a	bc	c				
Haematocrit	ns	< 0.001	0.014	ns	ns	ns	ns					*	#	B	A
Haemoglobin	< 0.001	ns	ns	ns	0.021	ns	ns								
MCV	< 0.001	ns	ns	ns	0.05	ns	ns								
MCH	< 0.001	ns	ns	ns	ns	ns	ns	a	b	a	a				
MCHC	< 0.001	ns	0.008	ns	ns	ns	ns	a	b	a	a			A	B

Factorial ANOVA	Time × Diet							
	CTRL				TRP			
Haematological parameters	1w	2w	3w	4w	1w	2w	3w	4w
WBC								
RBC								
Haematocrit								
Haemoglobin	a	Ab	a	a	a	Bb	a	a
MCV	ab	b	ab	a	a	b	a	a
MCH								
MCHC								

Values represent means ± SD (n = 6). Different symbols stand for statistically significant differences attributed to stimulation (*<#). Low case letters stand for statistically significant differences attributed to sampling time (a<b). Capital letters stand for statistically significant differences attributed to dietary treatment (A<B). (Multifactorial ANOVA; Tukey post-hoc test; ns: non-significant; $P \leq 0.05$).

Table S2. Peripheral leucocyte counts of European seabass fed dietary treatments and sampled at 1, 2, 3 and 4 weeks post injection.

Peripheral leucocytes	Dietary treatment	1 week		2 weeks		3 weeks		4 weeks	
		HBSS	FIA	HBSS	FIA	HBSS	FIA	HBSS	FIA
Neutrophils ($\times10^4$ μl^{-1})	CTRL	0.12 ± 0.07	0.27 ± 0.06	0.00 ± 0.00	0.10 ± 0.06	0.09 ± 0.10	0.13 ± 0.11	0.00 ± 0.00	0.04 ± 0.01
	TRP	0.11 ± 0.03	0.23 ± 0.12	0.04 ± 0.04	0.02 ± 0.03	0.09 ± 0.06	0.09 ± 0.06	0.19 ± 0.20	0.02 ± 0.02
Monocytes ($\times10^4$ μl^{-1})	CTRL	0.22 ± 0.16	0.61 ± 0.38 a	1.22 ± 0.84	1.28 ± 0.39 Aa	1.21 ± 0.67 *	2.61 ± 1.24 #Bb	0.69 ± 0.32	1.31 ± 0.41 a
	TRP	0.14 ± 0.05 a	0.20 ± 0.06 a	1.74 ± 0.14 *b	3.59 ± 1.02 #Bb	1.08 ± 0.80 ab	1.08 ± 0.79 Aa	0.52 ± 0.22 ab	1.53 ± 0.55 a
Lymphocytes ($\times10^4$ μl^{-1})	CTRL	2.20 ± 0.51	4.04 ± 0.57	1.53 ± 0.16	3.53 ± 0.76	1.50 ± 0.25	2.03 ± 0.89	1.44 ± 0.22	2.20 ± 0.21
	TRP	2.50 ± 0.16	4.13 ± 0.93	1.83 ± 0.19	2.80 ± 0.38	1.66 ± 0.66	1.66 ± 0.66	2.13 ± 0.70	3.54 ± 1.72
Thrombocytes ($\times10^4$ μl^{-1})	CTRL	3.02 ± 0.60	3.73 ± 0.55	2.65 ± 0.35	3.53 ± 0.58	2.88 ± 0.47	3.92 ± 0.81	4.18 ± 0.89	3.75 ± 0.50 A
	TRP	3.28 ± 0.51 ab	3.03 ± 0.08 a	2.16 ± 0.36 a	3.48 ± 1.32 a	3.56 ± 0.70 ab	3.56 ± 0.70 a	4.65 ± 1.19 *b	6.47 ± 1.42 #Bb

Factorial ANOVA	P-value							Time × Stimulus							
								HBSS				FIA			
Peripheral leucocytes	Time	Stimulus	Diet	Time × Stimulus	Time × Diet	Diet × Stimulus	Time × Diet × Stimulus	1w	2w	3w	4w	1w	2w	3w	4w
Neutrophils	< 0.001	0.04	ns	0.004	0.04	0.02	ns	*				#b	a	a	a
Monocytes	< 0.001	< 0.001	ns	ns	< 0.001	ns	0.005								
Lymphocytes	< 0.001	< 0.001	ns	0.008	ns	ns	ns	*	*		*	#c	#bc	a	#b
Thrombocytes	< 0.001	< 0.001	ns	ns	< 0.001	ns	0.003								

Factorial ANOVA	Time × Diet								Diet × Stimulus			
	CTRL				TRP				CTRL		TRP	
Peripheral leucocytes	1w	2w	3w	4w	1w	2w	3w	4w	HBSS	FIA	HBSS	FIA
Neutrophils	b	a	ab	a	b	a	a	ab	*	#		
Monocytes												
Lymphocytes												
Thrombocytes												

Values represent means ± SD (n = 6). Different symbols stand for statistically significant differences attributed to stimulation (*<#). Low case letters stand for statistically significant differences attributed to sampling time (a<b). Capital letters stand for statistically significant differences attributed to dietary treatment (A<B). (Multifactorial ANOVA; Tukey post-hoc test; ns: non-significant; P ≤ 0.05).

Table S3. Peritoneal leucocyte counts of European seabass fed dietary treatments and sampled at 1, 2, 3 and 4 weeks post injection.

Peritoneal leucocytes	Dietary treatment	1 week		2 weeks		3 weeks		4 weeks	
		HBSS	FIA	HBSS	FIA	HBSS	FIA	HBSS	FIA
Total peritoneal WBC ($\times 10^4$ μl^{-1})	CTRL	0.41 ± 0.25	1.91 ± 0.22	0.31 ± 0.11	2.18 ± 0.74	0.16 ± 0.04	1.75 ± 0.85	0.25 ± 0.04	2.57 ± 1.40
	TRP	0.51 ± 0.28	2.03 ± 0.95	0.50 ± 0.18	1.81 ± 0.61	0.21 ± 0.10	2.45 ± 1.24	0.25 ± 0.12	2.96 ± 1.88
Macrophages ($\times 10^4$ μl^{-1})	CTRL	0.04 ± 0.01	0.85 ± 0.10	0.10 ± 0.05	1.57 ± 0.52	0.06 ± 0.03	1.24 ± 0.31	0.08 ± 0.05	1.73 ± 1.07
	TRP	0.11 ± 0.06	0.78 ± 0.51	0.22 ± 0.03	1.33 ± 0.42	0.11 ± 0.01	1.38 ± 0.63	0.10 ± 0.08	1.54 ± 1.14
Neutrophils ($\times 10^4$ μl^{-1})	CTRL	0.07 ± 0.04	0.82 ± 0.07	0.07 ± 0.04	0.39 ± 0.22	0.05 ± 0.03	0.28 ± 0.16	0.06 ± 0.03	0.34 ± 0.24
	TRP	0.10 ± 0.07	0.89 ± 0.26	0.09 ± 0.06	0.31 ± 0.18	0.04 ± 0.01	0.28 ± 0.23	0.05 ± 0.03	0.25 ± 0.12
Lymphocytes ($\times 10^4$ μl^{-1})	CTRL	0.18 ± 0.11	0.34 ± 0.06	0.14 ± 0.07	0.20 ± 0.03	0.06 ± 0.01	0.28 ± 0.10	0.06 ± 0.01	0.52 ± 0.24
	TRP	0.24 ± 0.16	0.41 ± 0.11	0.18 ± 0.04	0.16 ± 0.06	0.08 ± 0.07	0.45 ± 0.17	0.13 ± 0.02	0.68 ± 0.28

Factorial ANOVA	P-value							Stimulus		Time × Stimulus							
										HBSS				FIA			
Peritoneal leucocytes	Time	Stimulus	Diet	Time × Stimulus	Time × Diet	Diet × Stimulus	Time × Diet × Stimulus	HBSS	FIA	1w	2w	3w	4w	1w	2w	3w	4w
Total peritoneal WBC	ns	< 0.001	ns	ns	ns	ns	ns	*	#								
Macrophages	ns	< 0.001	ns	ns	ns	ns	ns	*	#								
Neutrophils	< 0.001	< 0.001	ns	< 0.001	ns	ns	ns			*	*	*	*	#b	#a	#a	#a
Lymphocytes	<0.001	< 0.001	ns	< 0.001	ns	ns	ns					*	*	b	a	#b	#c

Values represent means ± SD (n = 6). Different symbols stand for statistically significant differences attributed to stimulation (*<#). Low case letters stand for statistically significant differences attributed to sampling time (a<b). Capital letters stand for statistically significant differences attributed to dietary treatment (A<B). (Multifactorial ANOVA; Tukey post-hoc test; ns: non-significant; P ≤ 0.05).

Table S4. Humoral parameters in European seabass fed dietary treatments and sampled at 1, 2, 3 and 4 weeks post injection.

Humoral parameters	Dietary treatment	1 week		2 weeks		3 weeks		4 weeks	
		HBSS	FIA	HBSS	FIA	HBSS	FIA	HBSS	FIA
Cortisol (ng ml^{-1})	CTRL	50.6 ± 59.8	8.1 ± 2.3	121.7 ± 130.4	71.4 ± 35.7	163.6 ± 132.7	156.6 ± 90.1	41.3 ± 28.7	33.8 ± 23.9
	TRP	88.3 ± 104.7	45.9 ± 13.4	52.9 ± 45.2	86.1 ± 58.8	149.9 ± 117.5	103.9 ± 60.8	18.4 ± 11.8	12.8 ± 10.9
Peroxidase (U ml^{-1})	CTRL	82.5 ± 31.3	65.0 ± 11.6	85.3 ± 57.5	26.4 ± 34.3	40.7 ± 33.2	50.3 ± 44.5	21.1 ± 21.2	13.4 ± 10.3
	TRP	33.9 ± 29.9	117.5 ± 37.5	37.1 ± 22.0	47.4 ± 41.4	43.3 ± 46.5	60.2 ± 66.5	26.8 ± 24.9	7.5 ± 1.0
Lysozyme activity (µg ml^{-1})	CTRL	22.5 ± 8.6	18.2 ± 12.7	18.4 ± 6.4	17.3 ± 1.5	16.7 ± 3.3	29.0 ± 1.2	16.9 ± 0.9	26.0 ± 8.1
	TRP	25.7 ± 14.9	11.6 ± 1.9	21.0 ± 1.8	22.3 ± 4.5	22.1 ± 3.0	24.2 ± 4.6	16.9 ± 3.1	35.8 ± 3.6
Total bactericidal activity (%)	CTRL	28.1 ± 27.4	23.1 ± 5.5	7.6 ± 5.7	15.1 ± 0.2	12.7 ± 2.3	13.5 ± 10.2	15.8 ± 6.2	5.7 ± 0.2
	TRP	15.5 ± 3.2	17.1 ± 4.3	3.1 ± 2.4	3.0 ± 0.7	14.1 ± 2.7	7.9 ± 5.9	12.8 ± 3.4	3.8 ± 3.3

Factorial ANOVA	P-value							Time				Diet	
Humoral parameters	Time	Stimulus	Diet	Time × Stimulus	Time × Diet	Diet × Stimulus	Time × Diet × Stimulus	1w	2w	3w	4w	CTRL	TRP
Cortisol	< 0.001	ns	ns	ns	ns	ns	ns	a	a	b	a		
Peroxidase	< 0.001	ns	ns	ns	ns	ns	ns	b	ab	ab	a		
Lysozyme	ns	ns	ns	< 0.001	ns	ns	ns						
Total bactericidal activity	< 0.001	ns	0.02	ns	ns	ns	ns	b	a	a	a	B	A

Factorial ANOVA	Time × Stimulus							
	HBSS				FIA			
Humoral parameters	1w	2w	3w	4w	1w	2w	3w	4w
Cortisol								
Peroxidase								
Lysozyme				*	a	ab	bc	# c
Total bactericidal activity								

Values represent means ± SD (n = 6). Different symbols stand for statistically significant differences attributed to stimulation (*<#). Low case letters stand for statistically significant differences attributed to sampling time (a<b). Capital letters stand for statistically significant differences attributed to dietary treatment (A<B). (Multifactorial ANOVA; Tukey post-hoc test; ns: non-significant; P ≤ 0.05).

Table S5. Immune and oxidative stress parameters in the gut of European seabass fed dietary treatments and sampled at 1, 2, 3 and 4 weeks post injection.

Gut parameters	Dietary treatment	1 week		2 weeks		3 weeks		4 weeks	
		HBSS	FIA	HBSS	FIA	HBSS	FIA	HBSS	FIA
Superoxide dismutase (U mg^{-1} protein)	CTRL	125.7 ± 36.8	130.2 ± 24.4	134.7 ± 33.3	251.1 ± 53.6	140.3 ± 7.6	325.0 ± 114.4	167.1 ± 89.7	122.4 ± 45.6
	TRP	119.0 ± 18.1	199.3 ± 15.3	148.7 ± 7.0	280.0 ± 121.8	118.4 ± 16.0	263.8 ± 97.9	168.8 ± 38.4	134.5 ± 6.1
Catalase activity (U mg^{-1} protein)	CTRL	27.9 ± 3.1	30.1 ± 9.6 a	29.2 ± 10.7 *	63.7 ± 6.4 #bc	38.2 ± 14.4 *	80.6 ± 21.6 #Bc	49.2 ± 11.3	44.3 ± 9.4 ab
	TRP	22.5 ± 3.7	42.1 ± 6.1	30.1 ± 1.4	55.9 ± 10.1	39.9 ± 5.0	53.1 ± 20.4 A	41.6 ± 13.4	71.6 ± 18.3
GSH/GSSG	CTRL	12.8 ± 1.6	7.9 ± 4.5	30.4 ± 26.7	8.4 ± 3.9	24.4 ± 22.7	31.5 ± 23.2	22.0 ± 24.3	19.3 ± 16.1
	TRP	13.8 ± 4.8	7.3 ± 2.2	37.9 ± 20.9	7.4 ± 5.5	16.5 ± 6.2	19.1 ± 3.4	10.4 ± 3.3	13.4 ± 2.5
Peroxidase (U ml^{-1})	CTRL	190.9 ± 40.7	83.2 ± 19.7	110.1 ± 21.2	79.5 ± 11.5	171.1 ± 23.9	116.7 ± 35.8	172.7 ± 43.5	146.5 ± 46.7
	TRP	153.3 ± 36.4	121.3 ± 21.7	83.3 ± 5.3	77.9 ± 8.0	164.3 ± 62.1	136.0 ± 63.6	179.6 ± 76.5	141.9 ± 30.7
Total bactericidal activity (%)	CTRL	61.2 ± 2.0 ab	55.2 ± 2.9 a	53.2 ± 8.6 Aa	54.9 ± 4.6 a	63.8 ± 1.5 ab	60.6 ± 6.5 ab	64.2 ± 2.7 b	68.1 ± 1.6 b
	TRP	59.0 ± 6.1	59.0 ± 10.0 ab	65.0 ± 3.0 B	54.0 ± 2.8 a	68.3 ± 2.5	65.6 ± 2.1 b	67.4 ± 3.3	67.1 ± 1.5 b

Factorial ANOVA	P-value							Time				Stimulus	
Gut parameters	Time	Stimulus	Diet	Time × Stimulus	Time × Diet	Diet × Stimulus	Time × Diet × Stimulus	1w	2w	3w	4w	HBSS	FIA
Superoxide dismutase	< 0.001	< 0.001	ns	< 0.001	ns	ns	ns						
Catalase activity	< 0.001	< 0.001	ns	0.04	0.048	ns	0.001						
GSH/GSSG	ns	ns	ns	0.013	ns	ns	ns						
Peroxidase	< 0.001	< 0.001	ns	ns	ns	ns	ns	b	a	b	b	#	*
Total bactericidal activity	< 0.001	ns	0.006	ns	ns	ns	0.03						

Factorial ANOVA	Time × Stimulus							
	HBSS				FIA			
Gut parameters	1w	2w	3w	4w	1w	2w	3w	4w
Superoxide dismutase		*	*		a	# b	# b	a
Catalase activity								
GSH/GSSG		#				*		
Peroxidase								
Total bactericidal activity								

Values represent means ± SD (n = 6). Different symbols stand for statistically significant differences attributed to stimulation (*<#). Low case letters stand for statistically significant differences attributed to sampling time (a<b). Capital letters stand for statistically significant differences attributed to dietary treatment (A<B). (Multifactorial ANOVA; Tukey post-hoc test; ns: non-significant; P ≤ 0.05).

Table S6. Gene expression in the head-kidney of European seabass fed dietary treatments and sampled at 1, 2, 3 and 4 weeks post injection.

Genes	Dietary treatment	1 week		2 weeks		3 weeks		4 weeks	
		HBSS	FIA	HBSS	FIA	HBSS	FIA	HBSS	FIA
gr1	CTRL	0.85 ± 0.25	0.48 ± 0.13	0.95 ± 0.16	0.86 ± 0.12	0.20 ± 0.09	0.23 ± 0.09	0.49 ± 0.08	0.45 ± 0.08
	TRP	0.94 ± 0.19	0.72 ± 0.26	0.81 ± 0.23	0.80 ± 0.22	0.22 ± 0.07	0.36 ± 0.14	0.43 ± 0.07	0.48 ± 0.05
mc2r	CTRL	2.32 ± 1.48	1.53 ± 0.95	1.11 ± 1.01	1.48 ± 0.94	0.61 ± 0.56	0.16 ± 0.11	0.49 ± 0.47	1.10 ± 0.93
	TRP	2.28 ± 1.29	1.92 ± 1.36	0.81 ± 0.67	1.28 ± 0.28	0.55 ± 0.42	0.78 ± 0.42	0.42 ± 0.34	0.42 ± 0.47
tcrα	CTRL	0.95 ± 0.34	0.71 ± 0.21	1.09 ± 0.09	0.92 ± 0.28	0.60 ± 0.19	0.72 ± 0.12	0.90 ± 0.53	0.82 ± 0.15
	TRP	1.00 ± 0.08	0.66 ± 0.12	0.90 ± 0.10	0.84 ± 0.32	0.48 ± 0.20	0.75 ± 0.25	0.73 ± 0.13	0.90 ± 0.11
ido2	CTRL	0.90 ± 0.18	0.79 ± 0.16	1.26 ± 0.16	0.72 ± 0.19	0.55 ± 0.08	0.67 ± 0.23	0.71 ± 0.37	0.98 ± 0.27
	TRP	0.92 ± 0.30	0.72 ± 0.22	0.99 ± 0.16	0.92 ± 0.33	0.41 ± 0.12	0.57 ± 0.18	0.59 ± 0.21	0.76 ± 0.31
Il1β	CTRL	0.11 ± 0.03 a	0.14 ± 0.02 b	0.11 ± 0.04 a	0.11 ± 0.02 ab	0.09 ± 0.03 a	0.07 ± 0.02 a	0.18 ± 0.04 b	0.12 ± 0.02 ab
	TRP	0.13 ± 0.04	0.12 ± 0.01	0.13 ± 0.02	0.12 ± 0.00	0.11 ± 0.04	0.09 ± 0.02	0.11 ± 0.04	0.14 ± 0.01
mcsfr	CTRL	0.66 ± 0.08	0.51 ± 0.10 a	0.67 ± 0.04	0.70 ± 0.07 a	0.67 ± 0.07	0.66 ± 0.19 a	0.77 ± 0.17	0.98 ± 0.12 b
	TRP	0.57 ± 0.12 a	0.54 ± 0.03 a	0.59 ± 0.03 *ab	0.87 ± 0.17 #b	0.57 ± 0.06 *ab	0.92 ± 0.26 #b	0.83 ± 0.06 b	0.90 ± 0.12 b
cxcr4	CTRL	0.67 ± 0.12 ab	0.70 ± 0.19 b	0.63 ± 0.15 ab	0.52 ± 0.08 ab	0.71 ± 0.17 Bb	0.49 ± 0.03 ab	0.47 ± 0.10 a	0.46 ± 0.06 a
	TRP	0.60 ± 0.13	0.71 ± 0.02 b	0.62 ± 0.12	0.63 ± 0.16 ab	0.42 ± 0.03 A	0.67 ± 0.08 b	0.41 ± 0.03	0.42 ± 0.07 a
il34	CTRL	0.81 ± 0.23 b	0.47 ± 0.11 A	0.51 ± 0.11 ab	0.59 ± 0.12	0.17 ± 0.01 a	0.33 ± 0.20	0.48 ± 0.13 ab	0.45 ± 0.11
	TRP	0.63 ± 0.08	0.82 ± 0.47 Bb	0.43 ± 0.07	0.43 ± 0.07a	0.25 ± 0.03	0.44 ± 0.06 ab	0.42 ± 0.15	0.60 ± 0.09 ab
tgfβ	CTRL	0.49 ± 0.08	0.48 ± 0.11	0.45 ± 0.02	0.50 ± 0.09	0.29 ± 0.00	0.31 ± 0.10	0.34 ± 0.06	0.36 ± 0.06
	TRP	0.46 ± 0.09	0.60 ± 0.06	0.41 ± 0.07	0.45 ± 0.09	0.19 ± 0.02	0.37 ± 0.11	0.31 ± 0.10	0.42 ± 0.04
il10	CTRL	1.24 ± 0.62	3.93 ± 2.64	1.07 ± 0.59	3.24 ± 1.33	0.92 ± 0.64	1.36 ± 0.75	0.58 ± 0.22	1.90 ± 0.49
	TRP	1.82 ± 0.89	3.75 ± 1.89	1.86 ± 0.54	2.69 ± 0.66	0.55 ± 0.28	1.09 ± 0.26	1.07 ± 0.71	1.41 ± 0.24

Factorial ANOVA	P-value							Time				Stimulus		Time × Stimulus							
														HBSS				FIA			
Genes	Time	Stimulus	Diet	Time × Stimulus	Time × Diet	Diet × Stimulus	Time × Diet × Stimulus	1w	2w	3w	4w	HBSS	FIA	1w	2w	3w	4w	1w	2w	3w	4w
gr1	ns	ns	ns	<0.001	0.036	ns	ns							#c	c	a	b	*b	c	a	ab
mc2r	<0.001	ns	ns	ns	ns	ns	ns	b	a	a	a										
tcrα	0.002	ns	ns	0.015	ns	ns	ns							b	b	a	ab				
ido2	<0.001	ns	ns	<0.001	ns	ns	ns							bc	c	a	ab				
Il1β	<0.001	ns	ns	ns	ns	ns	0.009														
mcsfr	<0.001	<0.001	ns	0.008	ns	0.008	0.014														
cxcr4	<0.001	ns	ns	ns	ns	<0.001	0.018														
il34	<0.001	ns	ns	ns	ns	0.02	0.018														
tgfβ	<0.001	<0.001	ns	ns	ns	ns	ns	b	b	a	a	*	#								
il10	<0.001	<0.001	ns	ns	ns	ns	ns	b	b	a	a	*	#								

Factorial ANOVA	Time × Diet							
	CTRL				TRP			
Genes	1w	2w	3w	4w	1w	2w	3w	4w
gr1	c	d	a	b	b	b	a	a
mc2r								
tcr								
ido2								
Il1β								
mcsfr								
cxcr4								
il34								
tgfβ								
il10								

Values represent means ± SD (n = 9). Different symbols stand for statistically significant differences attributed to stimulation (*<#). Low case letters stand for statistically significant differences attributed to sampling time (a<b). Capital letters stand for statistically significant differences attributed to dietary treatment (A<B). (Multifactorial ANOVA; Tukey post-hoc test; ns: non-significant; P ≤ 0.05).
